# Dissolved oxygen limitation and *Pythium* root rot in strawberry NFT systems: mechanisms, research gaps, and prospects for substrate-free production

**DOI:** 10.3389/fpls.2026.1829367

**Published:** 2026-05-13

**Authors:** Shalyne Scott, Camilo Villouta

**Affiliations:** Controlled Environment Agriculture Lab, Department of Plant Sciences and Entomology, University of Rhode Island, Kingston, RI, United States

**Keywords:** controlled environment agriculture, dissolved oxygen, *Fragaria × ananassa*, nutrient film technique, *Pythium* root rot, recirculating hydroponic systems, rhizosphere hypoxia, zoospore dispersal

## Abstract

Strawberry (*Fragaria* × *ananassa* Duch.) production faces growing pressure to reduce reliance on peat and coconut coir substrates, driven by documented life cycle liabilities including carbon losses from peat extraction and embodied transport emissions from coir. Nutrient film technique (NFT), a substrate-free recirculating hydroponic system, eliminates growing media entirely and reduces material inputs across successive crop cycles, making it an environmentally attractive candidate for controlled environment strawberry production. Despite early commercial adoption in Europe during the 1970s, NFT was largely abandoned for strawberry production by the 1980s following systematic failures whose physiological basis remains incompletely characterized. This review synthesizes evidence from hydroponic systems engineering, plant physiology, and oomycete pathology to examine the two structural constraints underlying NFT’s historical rejection: dissolved oxygen depletion dynamics within recirculating nutrient solution, and exceptional susceptibility to *Pythium* spp. root rot. We demonstrate that these constraints are coupled rather than independent, sharing a common pathway through root-zone oxygen status. Progressive root mat development over a six-month fruiting cycle degrades passive film aeration and creates hypoxic conditions that impair root membrane integrity, alter rhizosphere exudate profiles, and facilitate *Pythium* zoospore encystment and necrotrophic transition. This interaction is compounded by strawberry’s exceptional oxygen sensitivity and absence of adaptive aerenchyma formation, rendering thresholds established for tomato and cucumber inapplicable to this species. We identify two prerequisite research gaps that must be resolved before NFT can be rationally reconsidered for commercial strawberry production: characterization of root mat effects on channel hydraulic performance, and establishment of a strawberry-specific dissolved oxygen threshold under NFT-relevant conditions.

## 1 Introduction

Strawberry (*Fragaria* × *ananassa*) consumption continues to expand, driven by its increasing recognition for nutritional, economic, and ecological significance among consumers, growers, and researchers. This demand is supported by strawberries desirable flavor, aroma, and visual appeal, as well as their year-round availability (Samtani et al., 2019). In addition to their economic importance, strawberries are rich in bioactive compounds, including flavonoids and phenolic acids, which contribute to their antioxidant capacity and associated health benefits (Giampieri et al., 2012). Beyond fresh consumption, strawberries are widely incorporated into processed foods, displaying remarkable versatility in both domestic and industrial applications (Ibrahim et al., 2023). In 2023, the U.S. strawberry industry was valued at $3.39 billion (USDA National Agricultural Statistics Service, 2022), with California being the top producer and Florida ranking second in overall production (Clark and Mousavi-Avval, 2022; Hernández-Martínez et al., 2023; Holmes, 2024; Samtani et al., 2019). Nearly all California and Florida strawberry production occurs on raised beds covered with plastic film (Holmes, 2024; Sideman and Orde, 2023). The annual hill or plasticulture system is the dominant U.S. strawberry cultivation method, consisting of raised beds covered with plastic mulch and drip irrigation, which optimize soil moisture retention, reduce weed infestations, and prevent fruit-soil contact, thereby ensuring profitable yields (Black et al., 2002; Hancock and Simpson, 1995; Samtani et al., 2019; Sideman and Orde, 2023; Voth and Bringhurst, 1990; Weber, 2021a, b). However, this system comes with high initial costs, produces only one crop per year, relies heavily on fumigants and mulches to manage soilborne diseases, and carries an increased risk of frost and winter injury (Johnson and Hoffmann, 2024; Sideman and Orde, 2023).

As an alternative, Controlled Environment Agriculture (CEA) systems are gaining popularity in strawberry production (Samtani et al., 2019). CEA is a technology-driven approach that optimizes plant growth by precisely controlling temperature, humidity, lighting, CO_2_ levels, and nutrient delivery, enabling year-round production independent of regional climatic constraints while enhancing yield, fruit quality, and resource efficiency (Darby and Islam, 2025; Gómez et al., 2019; Lu et al., 2023; Nichols, 2015; Rouphael et al., 2018). Recent advances in automation and artificial intelligence (AI) have further enhanced CEA systems, enabling real-time monitoring, predictive modeling, and dynamic optimization of environmental conditions, improving resource use efficiency and crop performance (Fuentes-Peñailillo et al., 2024; Lephondo et al., 2024; Rouphael et al., 2018). CEA labor costs are often lower than traditional farming, accounting for 20-30% of total production costs, as ergonomic designs improve harvesting efficiency (Demircan et al., 2019; Hernández-Martínez et al., 2023; Paranjpe et al., 2004). While CEA systems can improve labor efficiency and resource use, total production costs are not consistently lower than conventional systems due to higher initial investment and energy requirements (Pomoni et al., 2023). Nonetheless, they provide greater production stability through precise control of environmental conditions that regulate temperature, light, humidity, and nutrient delivery (Gómez et al., 2019; Samtani et al., 2019).

In California strawberry cultivation specifically, tabletop production has gained considerable popularity as an alternative to in-soil systems (Samtani et al., 2019; Van Delm et al., 2016). The term encompasses any hydroponic system elevated to table height, including vertical towers, A-frame planters, gutter systems, and grow bags on benches (Fessler et al., 2022; Kouloumprouka Zacharaki et al., 2024; Lieten, 1998; Lieten and Baets, 1991; Lieten and Marcelle, 1993). These systems use soilless substrates that eliminate the need for crop rotation and fumigation and are replaced annually (Milinkovic et al., 2015). The two most widely used substrates are coconut coir and peat-based mixes, each offering distinct agronomic trade-offs in porosity, pH stability, sustainability, and cost (Carrijo et al., 2002; Meerow, 1994; Yafuso and Boldt, 2024).

Between 2009 and 2019, U.S. hydroponic strawberry production surged from 5.94 to 83.32 metric tons as protected cultivation areas expanded (USDA National Agricultural Statistics Service, 2009, 2019). Among the hydroponic systems available for commercial strawberry production, drip-substrate systems dominate, while Nutrient Film Technique (NFT) remains infrequently used (Hutchinson et al., 2025). NFT is a water culture technique in which bare-rooted plants are sustained by a thin, continuously recirculating nutrient solution flowing through horizontal channels or troughs (Morgan, 2006; Tüzel et al., 2019; Van Delm et al., 2016). The system offers meaningful advantages: it eliminates substrate entirely, removing soil-related constraints such as erosion, compaction, nematodes, and salinity accumulation; it conserves water and fertilizer through recirculation; and it can deliver nutrients directly to roots with minimal overhead (Gómez et al., 2019; Graves, 1983). NFT’s substrate-free design reduces both material inputs and system mass, features that make it particularly tractable in space-limited or urban production contexts (Graves, 1983; Palmitessa et al., 2024; Wheeler, 2017).

The economic and environmental significance of substrate elimination extends beyond the agronomic advantages of NFT. In commercial greenhouse strawberry production, substrate represents a recurring annual input that must be sourced, transported, installed, and disposed of at the end of each six-month crop cycle, with supply chain disruptions and long transportation distances from production regions in Europe and Asia contributing substantially to both input cost and environmental footprint for North American growers (Gruda, 2019; Stoessel et al., 2012; Woznicki et al., 2024). The two materials that dominate current practice, European block peat and coconut coir, each carry documented environmental burdens. Life cycle assessment confirms that peat extraction permanently removes carbon from peatland ecosystems that function as long-term carbon sinks and generates persistent greenhouse gas emissions at extraction sites for decades, a consequence that has driven policy-driven phase-out commitments in several European countries (Bonn et al., 2014; Gruda, 2019; Stichnothe, 2022). Coconut coir, widely adopted as the more sustainable alternative, carries its own footprint: production involves land use change, water pollution during processing, and a substantial embodied CO_2_ cost from long-distance transport from South and Southeast Asia, and these concerns have been documented specifically in the context of greenhouse strawberry substrate selection (Gruda, 2019; Woznicki et al., 2024). A substrate-free production system eliminates this input category entirely, and the growing policy and market pressure against peat-based growing media reinforces the relevance of water-culture alternatives for operations seeking to reduce their environmental footprint over successive crop cycles.

Despite these advantages, NFT is rarely adopted for strawberry cultivation. The system was originally designed for short-cycle, non-fruiting crops with growth windows of 30 to 50 days, for which passive film aeration and limited buffering capacity are adequate (Burrage, 1992; Resh, 2022). Strawberry, by contrast, requires six months or more of continuous production, during which expanding root systems progressively fill narrow channels, increasing the risk of oxygen and nutrient deficiencies, while the absence of a substrate buffer amplifies vulnerability to solution interruptions and pathogen pressure (Fessler et al., 2022; Suhl et al., 2019). Wate source quality imposes a further system-level constraint in NFT that is absent from substrate-based systems: without a physical growing medium to buffer ion accumulation, elevated sodium or bicarbonate concentrations in the supply water are delivered directly and continuously to the root zone, where sodium competitively inhibits potassium and calcium uptake while chloride suppresses nitrate absorption through ion antagonism (Sambo et al., 2019). In recirculating closed-loop systems, these ions accumulate progressively across the crop cycle as they are not taken up proportionally by the plant, increasing solution salinity and compounding osmotic stress on roots already operating under limited oxygen supply (Fathidarehnijeh et al., 2023; Resh, 2022).

Root rot diseases are particularly problematic under the continuously moist, recirculating conditions of NFT because the shared nutrient solution connects all plants to a single continuous dispersal pathway: a single inoculum introduction anywhere in the recirculating loop provides direct hydraulic access to the entire crop simultaneously (Sutton et al., 2006; Postma et al., 2008). Unlike substrate-based systems, NFT channels lack both the soil microbial community and the physical filtration that buffer inoculum load before it reaches root surfaces (Paulitz, 1997). Primary inoculum enters systems through multiple routes: infected or symptomless transplants are among the most commonly documented vectors in commercial hydroponic production (Hong and Moorman, 2005; Amrhein et al., 2025), while contaminated source water, tools, and footwear represent additional entry pathways (Moorman et al., 2002; Hong and Moorman, 2005). Once established, system infrastructure including channels, pipes, and reservoir tanks functions as a persistent inoculum reservoir capable of initiating disease in successive crop cycles if not adequately sanitized between plantings (Postma et al., 2008; Sutton et al., 2006). These compounding limitations drove the abandonment of NFT for strawberry production in the United Kingdom, Belgium, and the Netherlands during the 1980s, following its introduction in the 1970s, with growers transitioning to peat and coir substrates that remain standard practice today (Van Delm et al., 2016).

**Figure 1:**
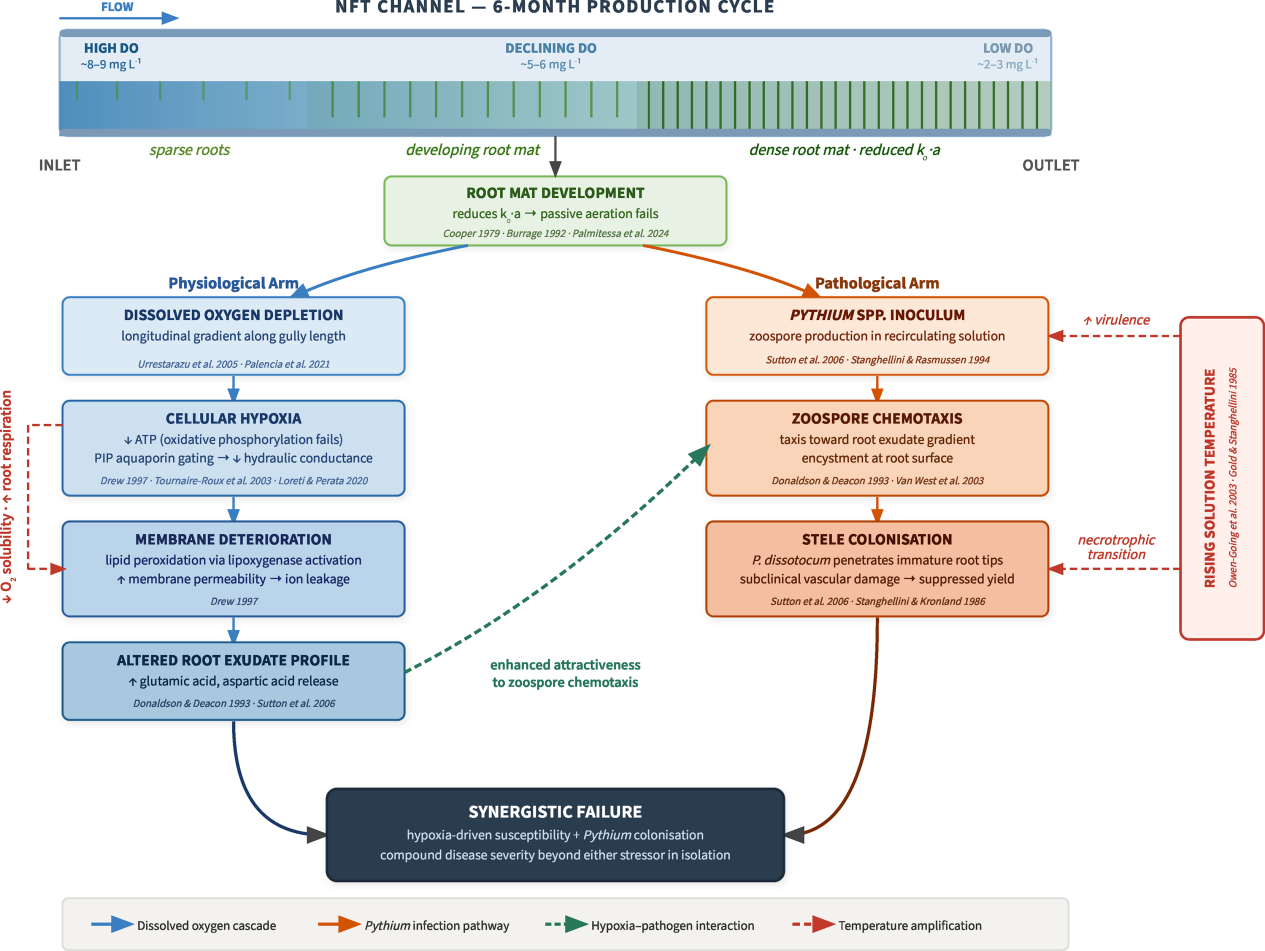
Coupled failure pathway of dissolved oxygen depletion and *Pythium* root rot in strawberry nutrient film technique (NFT) systems. The upper panel depicts the NFT channel across a six-month production cycle, illustrating the progressive development of root mat density from inlet to outlet and the corresponding longitudinal decline in dissolved oxygen (DO) concentration. Root mat accumulation degrades the gas–liquid interfacial area (k·a) governing passive film aeration, driving DO below physiologically critical thresholds at distal channel positions. The cascade bifurcates into two interacting arms. The physiological arm (left, blue) traces the consequences of root-zone hypoxia: ATP depletion via failure of oxidative phosphorylation, closure of plasma membrane intrinsic protein (PIP) aquaporins reducing hydraulic conductance, and lipid peroxidation via lipoxygenase activation that increases membrane permeability. The resulting shift in root exudate composition (elevated glutamic and aspartic acid release) directly enhances zoospore chemotactic attraction to the rhizosphere (dashed green arrow), linking the physiological arm to the pathological arm (right, orange). The pathological arm traces *Pythium* spp. inoculum dynamics in the recirculating solution, zoospore chemotaxis and encystment at compromised root surfaces, and stele colonization by *P. dissotocum* at immature root tips, producing subclinical vascular damage that suppresses marketable yield before above-ground symptoms appear. Rising solution temperature (dashed red) acts as a compound amplifier across both arms, simultaneously reducing oxygen solubility and root-zone oxygen availability, increasing pathogen virulence, and triggering the biotrophic-to-necrotrophic transition in already-colonized tissue. Both arms converge on a synergistic failure outcome whose severity exceeds what either stressor would produce in isolation, providing the mechanistic basis for the historical commercial failure of NFT in strawberry production.

The case for revisiting NFT in strawberry production rests on two converging pressures. Growing demand for resource-efficient, space-saving, and fumigant-free production methods has increased interest in water culture systems, while advances in root-zone oxygenation, pathogen management, and system design have materially changed what is technically achievable relative to the conditions under which NFT was abandoned four decades ago (Paranjpe et al., 2004; Samtani et al., 2019). This review examines the two primary constraints that historically limited NFT viability for strawberry, dissolved oxygen management and *Pythium* root rot (**Figure 1**), synthesizing current mechanistic understanding of each, identifying the specific research gaps that remain unresolved, and evaluating whether advances in science and system design provide a credible path toward making media-free NFT a viable option for long-cycle fruiting crop production.

## 2 Traditional, field cultivation vs CEA:

Conventional U.S. strawberry production relies primarily on the plasticulture system, in which annual raised beds with plastic mulch and drip irrigation optimize moisture retention and weed suppression but restrict output to a single crop per year, require substantial fumigant and labor inputs, and expose plants to climate-driven yield variability (Black et al., 2002; Johnson and Hoffmann, 2024; Samtani et al., 2019; Sideman and Orde, 2023). These structural constraints, combined with increasing regulatory pressure on soil fumigants and growing demand for year-round domestic supply, have accelerated grower interest in CEA systems capable of decoupling production from seasonal and regional climatic limitations (Paranjpe et al., 2004; Samtani et al., 2019).

CEA enables continuous production by maintaining temperature, humidity, CO_2_, photoperiod, and nutrient delivery within the narrow ranges strawberries require, collectively enabling yield stability not achievable in traditional field production (Gómez et al., 2019; Rouphael et al., 2018).

Within CEA, soilless substrate-based systems, principally tabletop and gutter configurations using coconut coir or peat, have become the commercial standard in North America and Europe (Samtani et al., 2019; Van Delm et al., 2016). Their dominance reflects a practical advantage: the substrate matrix provides root-zone buffering for moisture, oxygen, and nutrients that insulates the crop against transient system variability across the six-month production cycle. Water-culture systems, including NFT and deep-water culture, offer potential advantages in water and fertilizer efficiency and system cleanliness, but direct comparative trials have consistently found that substrate systems outperform water-culture configurations in strawberry yield and plant establishment under commercial conditions (Hutchinson et al., 2025). The mechanisms underlying this performance gap, specifically the structural inability of media-free NFT to maintain adequate dissolved oxygen supply and its vulnerability to *Pythium* root rot under long-cycle production conditions, are the subjects of the two sections that follow.

While conventional open-field strawberry production faces a broad spectrum of pathogens including Verticillium dahliae, Phytophthora cactorum, Macrophomina phaseolina, Colletotrichum spp., Botrytis cinerea, and multiple Pythium spp., as well as soil-transmitted viruses, many of which historically required chemical fumigation to suppress (Samtani et al., 2019; Hernández-Martínez et al., 2023), field systems also present significant risks from pathogen dissemination through plant material. This vulnerability is especially important in the California strawberry industry, where propagation still depends largely on open-field nursery systems rather than fully protected environments (Samtani et al., 2019; Shan et al., 2026). Evidence from these nursery systems shows that transplants can act as symptomless carriers of pathogens, facilitating disease spread across production regions and contributing to outbreaks in fruiting fields (Shan et al., 2026). Given the scale of the industry, which produces over 1.5 billion plants annually, even low levels of infection can generate substantial downstream impacts on crop health and yield (Holmes, 2024). In contrast, CEA systems can reduce initial exposure to certain soilborne pathogens through soilless production and greater environmental protection, but they create a distinct and narrower disease profile dominated by waterborne oomycetes; in recirculating water culture systems, Pythium spp. represent the primary biotic threat, capable of dispersing through the nutrient solution and causing system-wide losses with a speed and scale that has no equivalent in field or substrate-based production (Stanghellini and Rasmussen, 1994; Sutton et al., 2006; Amrhein et al., 2025). These differences highlight a fundamental trade-off between production systems, where CEA offers improved environmental control and potential reductions in initial pathogen exposure, but also requires substantially greater capital investment in climate control and recirculating system infrastructure than conventional field operations (Pomoni et al., 2023; Verteramo Chiu et al., 2024).

## 3 Dissolved oxygen limitations

### 3.1 Introduction and scope

NFT channels deliver nutrients through a thin, gravity-driven film of recirculating solution flowing across bare roots at approximately 1 L min^-^¹ per channel, with channel slopes of 1–3% and lengths typically limited to 10–15 m to prevent distal nutrient and oxygen depletion (Cooper, 1979; Graves, 1983). In Nutrient Film Technique systems, dissolved oxygen reaches plant roots exclusively through passive diffusion across the gas-liquid interface of a thin, gravity-driven film, a mechanism governed by the liquid-side mass transfer coefficient (Higbie, 1935) and specific interfacial area (Danckwerts, 1951) that is structurally unlike the gas-filled pore continuum of substrate-based systems or the forced aeration typical of deep water culture (Cooper, 1979; Graves, 1983). Because no substrate matrix retains oxygen between flow cycles and no supplemental aeration acts directly on the root zone, dissolved oxygen supply in NFT is entirely dependent on film hydraulics: shallow, fast-moving films maximize the air-water interface and surface renewal rate, while root mat development, rising solution temperature, and increasing biological oxygen demand progressively degrade both (Jackson et al., 1984). In closed recirculating systems, these dynamics compound across the crop cycle; operational dissolved oxygen concentrations in hydroponic systems typically range from 6 to 8%, with a recommended minimum of 5 mg L^-^¹ in commercial production (Holderness and Pegg, 1986; Zinnen, 1988), yet empirical measurements in soilless strawberry production confirm that dissolved oxygen can decline by nearly 40% between winter and spring, driven by both rising solution temperature and increasing biological oxygen demand as root biomass expands across the crop cycle (Palencia et al., 2021), and that channel-length depletion gradients alone can reduce dissolved oxygen from 6.2 to 2.9 mg/L between inlet and outlet under standard NFT flow conditions (Urrestarazu et al., 2005). Biological oxygen demand from expanding root biomass and microbial communities accumulates across the crop cycle with no compensatory mechanism, compounding the structural limitations of passive film aeration (Sutton et al., 2006). Importantly, dissolved oxygen deficiency is not only a physiological constraint but also a critical predisposing factor for disease development, as hypoxic root conditions reduce plant defense capacity and favor infection by waterborne pathogens such as Pythium spp (Sutton et al., 2006).

**Figure 2:**
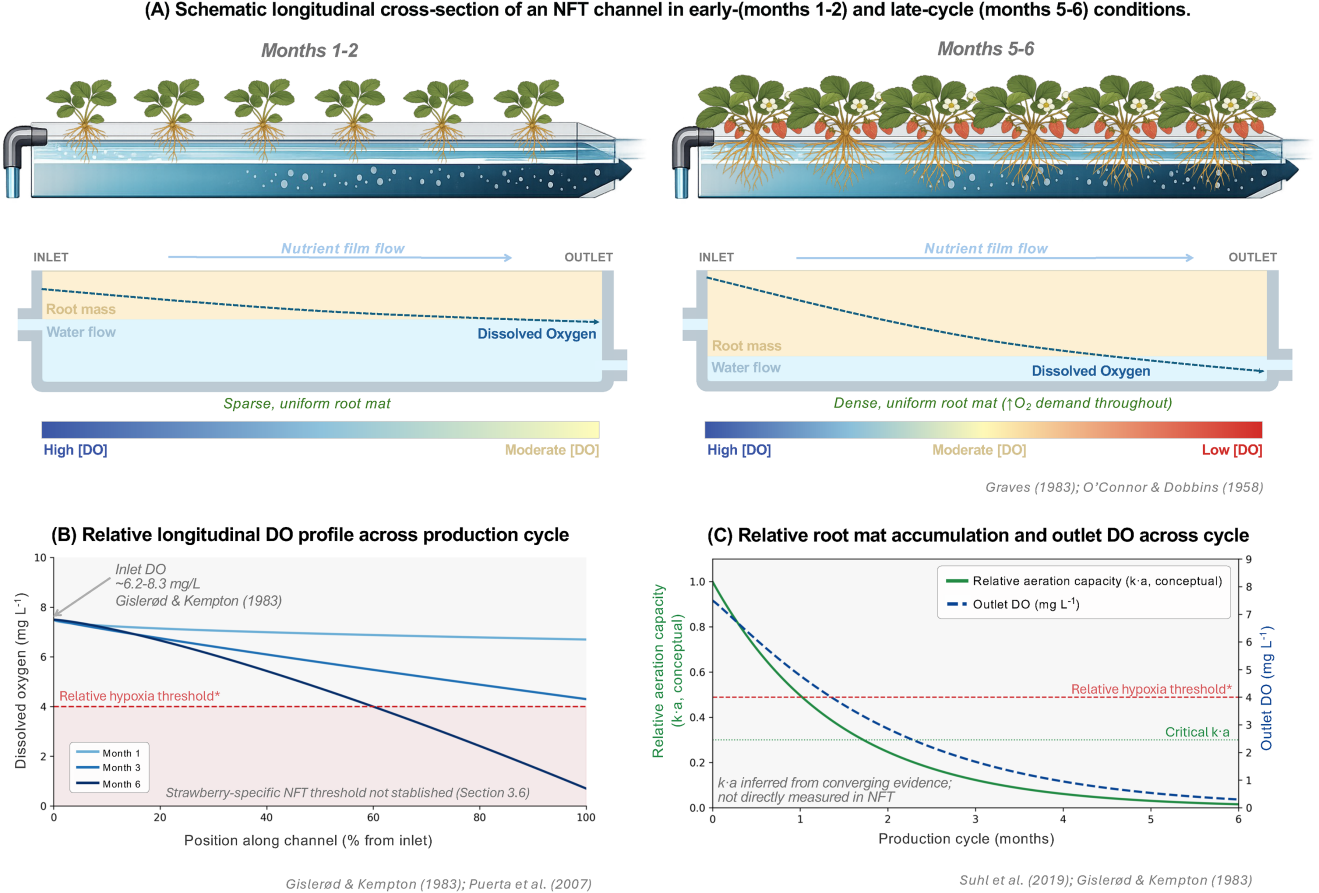
Spatial and temporal dynamics of dissolved oxygen depletion in strawberry NFT systems. **(A)** Schematic longitudinal cross-section of an NFT channel under early- (months 1–2) and late-cycle (months 5–6) conditions. A sparse root mat preserves adequate gas–liquid interfacial area (k·a) early in the cycle; a dense root mat increases oxygen demand throughout the channel by months 5–6, driving rapid DO decline toward the outlet. Root mat density gradient is conceptual. **(B)** Representative longitudinal DO profiles at months 1, 3, and 6. Inlet DO ~7.5 mg L^-^¹. Depletion at distal positions intensifies as root biomass accumulates. Dashed red line = relative hypoxia threshold (4 mg L^-^¹); a strawberry-specific DO threshold under NFT conditions has not been established (Section 3.6). Profiles are conceptual. **(C)** Conceptual temporal trajectories of relative aeration capacity (k·a, left axis) and outlet DO (right axis) across a six-month fruiting cycle. k·a is inferred from converging mechanistic and empirical evidence and has not been directly measured as a function of root mat accumulation in NFT systems.

Strawberry imposes a trajectory of oxygen demand on NFT that is structurally incompatible with the system as designed for short-cycle crops. Over a production cycle of six months or more, the crop develops a dense fibrous root mat that progressively reduces effective channel cross-section, increases local hydraulic resistance, and submerges root tissue that would otherwise remain in the air phase above the film, simultaneously degrading the mass transfer coefficient and the specific interfacial area available for gas exchange at precisely the period when respiratory demand peaks during fruit development (Burrage, 1992; Palencia et al., 2021; Palmitessa et al., 2024) (**Figure 2A**). Unlike flood-tolerant species capable of adaptive aerenchyma formation (Pedersen et al., 2021), strawberry possesses no internal oxygen-buffering mechanism, and all vascular continuity between root and shoot passes through a compact crown with no capacity to sustain aerobic metabolism during even moderate supply interruptions (Iwasaki, 2005; Morard and Silvestre, 1996). This combination of structural oxygen delivery deterioration and physiological oxygen sensitivity is the mechanistic basis for the historical failure of NFT in commercial strawberry production (**Figure 1**), and resolving it requires understanding each component of the chain with a precision that the current literature does not yet provide. Several strategies have been proposed to mitigate dissolved oxygen limitations in NFT and hydroponic systems, including supplementary oxygenation (Jackson et al., 1984; Palencia et al., 2021), modifications to channel design and flow dynamics (López-Pozos et al., 2011), and temperature regulation of nutrient solutions, which influences both oxygen availability and pathogen dynamics (Sutton et al., 2006).

This section examines that chain systematically. It reviews the physiological oxygen requirements of strawberry roots across the production cycle; the hydraulic, thermal, and biological drivers that shape dissolved oxygen behavior along NFT channels; the consequences of deficiency at the cellular, root-system, and whole-plant level; and the design and operational interventions that have been proposed or demonstrated to improve oxygenation in media-free systems. It concludes by identifying the two empirical gaps that currently prevent rational oxygen management in NFT strawberry production: the absence of characterized relationships between root mat development and hydraulic performance across commercially relevant channel geometries, and the absence of a strawberry-specific dissolved oxygen threshold established under NFT-relevant conditions.

### 3.2 Strawberry root-zone physiology and oxygen requirements

Unlike flood-tolerant species that form aerenchyma to facilitate internal oxygen diffusion under hypoxic conditions (Pedersen et al., 2021), strawberry shows rapid physiological deterioration under root-zone oxygen deficit, with no reported capacity for adaptive aerenchyma formation (Iwasaki, 2005; Morard and Silvestre, 1996). The cultivated strawberry (*Fragaria* × *ananassa*) possesses a shallow fibrous root system with all vascular connections to the shoot passing through a compact crown structure, making it one of the more oxygen-sensitive horticultural crops and distinguishing it fundamentally from flood-tolerant and even moderately tolerant species. This anatomical configuration means that any disruption to external oxygen supply propagates rapidly through the entire root system, with no internal buffering mechanism to sustain aerobic metabolism during periods of oxygen limitation.

The physiological requirement for oxygen in strawberry roots extends well beyond basic respiratory function: active mineral uptake depends on ATP generated through oxidative phosphorylation, meaning that even moderate oxygen limitation directly compromises nutrient absorption before any visible stress symptoms appear in the shoot (Drew, 1997).

Oxygen demand in strawberry roots is not constant across the production cycle; two stages are physiologically critical. During establishment, newly transplanted plants exhibit high respiratory activity as new roots develop and stored carbohydrates are rapidly consumed (Dickmann and Blanke, 2000). Any oxygen deficit at this stage can stunt early root development and delay or suppress subsequent flowering. During fruit development and filling, root oxygen demand increases again to sustain the active uptake of water and minerals required for berry enlargement; insufficient root-zone oxygen at this stage directly impairs nutrient transport and reduces marketable yield (Palencia et al., 2021; Steffens et al., 2005). In addition to its direct effects on root metabolism and nutrient uptake, dissolved oxygen availability plays a critical role in determining susceptibility to root pathogens in hydroponic systems (Sutton et al., 2006; Amrhein et al., 2025). Under hypoxic conditions, reduced ATP production and impaired membrane function weaken root integrity and overall physiological performance, limiting the plant’s capacity to resist infection (Drew, 1997; Morard and Silvestre, 1996). At the same time, low-oxygen conditions promote environments favorable for opportunistic, waterborne pathogens such as Pythium spp., which are well adapted to saturated and oxygen-limited root zones (Chérif et al., 1997; Sutton et al., 2006). This interaction between host stress and favorable pathogen conditions facilitates rapid colonization and disease development, positioning dissolved oxygen deficiency as a key predisposing factor for Pythium-associated root rot in NFT-grown strawberry systems (Sutton et al., 2006).

More recent work in soilless and controlled environment systems further supports the importance of root-zone oxygen supply in strawberry, demonstrating that modifications to oxygen availability can significantly influence root function, nutrient uptake, and yield responses (Palencia et al., 2021; Kaya et al., 2025), and comparative trials consistently find NFT underperforming substrate-based systems under commercial conditions, a pattern consistent with chronic oxygen limitation in media-free channels (Hutchinson et al., 2025). Direct dissolved oxygen threshold studies in strawberry NFT systems remain absent from the published literature, representing a critical gap that this review identifies as a prerequisite for rational system design.

Root-zone temperature exerts a strong and well-documented influence on strawberry root physiology independently of oxygen availability. Experimental work using a deep flow technique hydroponic system confirms that a root-zone temperature of 30 °C substantially decreases root oxygen consumption and cell viability in strawberry, ultimately causing plant wilting within two months of exposure (Sakamoto et al., 2016). Conversely, large diurnal fluctuations in root-zone temperature, even around a mean of 20 °C, reduce root dry weight, restrict nutrient uptake, and decrease fruit size independently of mean temperature effects (Gonzalez-Fuentes et al., 2016). These findings collectively indicate that root-zone thermal stability in the range of approximately 18 to 22 °C is necessary to maintain consistent root function in hydroponic strawberry production, and that temperature instability compounds any existing oxygen limitation by further impairing root cell viability and metabolic capacity.

Beyond the general impairment of active transport, oxygen deprivation produces specific nutrient imbalances with direct consequences for strawberry fruit quality. Calcium and boron, both elements with limited phloem mobility and therefore entirely dependent on continuous mass flow through the transpiration stream, are especially vulnerable to root-zone hypoxia; their deficiency in oxygen-poor environments contributes directly to tip burn and fruit deformities that reduce marketable quality (Drew, 1997; Steffens et al., 2005). Nitrogen dynamics are also specifically altered: root temperature and NH_4_^+^:NO_3_^-^ ratio interact to influence strawberry nitrogen absorption, with hypoxic and high-temperature conditions tending to impair nitrate assimilation, which requires substantial redox capacity and ATP supply in root cells (Ganmore‐Neumann and Kafkafi, 1985, 1983). An unmanaged shift in the nitrogen form balance under oxygen-limited conditions can therefore compound the primary stress with progressive secondary nutritional disorders.

While a specific dissolved oxygen threshold for strawberry root zones has not been rigorously established in the published literature, the cumulative evidence reviewed here indicates that oxygen management is a physiological necessity rather than a precautionary recommendation, one that NFT systems, with their reliance on passive film aeration alone, have historically struggled to meet under warm production conditions (Jackson et al., 1984; Morard and Silvestre, 1996).

### 3.3 Hypoxia

In NFT systems, dissolved oxygen can become progressively limiting as solution temperature rises, root oxygen demand increases, and microbial communities within the recirculating solution compete with roots as an additional oxygen sink (Jackson et al., 1984; Morard and Silvestre, 1996; Zeroni et al., 1983). These combined biological demands create localized hypoxia despite continuous solution flow, a dynamic that produces a consistent oxygen gradient along channel length, with concentrations at distal root positions capable of declining to physiologically critical levels under high crop demand (Gislerød and Kempton, 1983; Nitu et al., 2024). Rising solution temperature compounds this risk by simultaneously increasing root respiratory demand and reducing the oxygen-carrying capacity of water (Zheng et al., 2007). In soilless strawberry production specifically, Palencia et al. (2021) documented that dissolved oxygen in the drained fertigation solution dropped from 9.89 mg L^-^¹ in December to 5.99 mg L^-^¹ in April as solution temperature rose across the growing season, a decline of nearly 40% that coincided with measurable reductions in fruit yield, demonstrating that oxygen depletion dynamics are a measurable agronomic reality under standard soilless production conditions, not merely a theoretical concern.

As detailed in Section 3.2, hypoxia compromises ATP production through oxidative phosphorylation, directly impairing active nutrient absorption and membrane transport (Drew, 1997). To partially compensate, roots activate a conserved transcriptional response mediated by Ethylene Response Factor group VII (ERF-VII) proteins, which under low-oxygen conditions activate hypoxia-responsive genes encoding the fermentative enzymes pyruvate decarboxylase (PDC) and alcohol dehydrogenase (ADH) (Loreti and Perata, 2020; Van Veen et al., 2024). This shift to ethanolic fermentation sustains partial ATP production but at greatly reduced energetic efficiency, accelerating depletion of carbohydrate reserves in actively growing root tissues. It should be noted that this transcriptional and enzymatic response has been characterized primarily in model species; the degree to which strawberry roots activate an equivalent response under the moderate and fluctuating hypoxia typical of NFT conditions remains poorly documented, representing a meaningful gap in strawberry-specific stress physiology.

The consequences of root hypoxia are not confined to the root system but extend systemically to shoot function through impaired hydraulic conductance and hormonal signaling. Under oxygen deficiency, ATP depletion causes cytosolic acidification, which protonates a conserved histidine residue on the D-loop of plasma membrane intrinsic protein (PIP) aquaporins, physically closing the water channel pore and reducing root hydraulic conductivity (Tournaire-Roux et al., 2003; Kudoyarova et al., 2022); while this mechanism has not been directly validated in strawberry, the histidine gating site is conserved across angiosperms, making an analogous response in Fragaria × ananassa physiologically plausible (Kaya et al., 2025). The resulting decline in root hydraulic conductance, together with the energetic impairment of active water transport, limits water delivery to the shoot (Morard and Silvestre, 1996). In response to reduced shoot water status and root-derived chemical signals, stomatal conductance decreases, restricting intercellular CO_2_ availability and suppressing photosynthetic rate (Kaya et al., 2025; Toro et al., 2018). These shoot-level consequences have been directly documented in horticultural crops: in Capsicum, root hypoxia reduced root biomass, impaired leaf water uptake, decreased chlorophyll and carotenoid content, and significantly reduced the maximum quantum yield of photosystem II (Goto et al., 2022). While equivalent measurements have not been conducted in hydroponic strawberry under NFT-specific conditions, the mechanistic pathway from root oxygen deficit to shoot photosynthetic impairment is conserved across herbaceous crops.

Under hypoxic conditions, root metabolic disruption and membrane instability can alter exudation patterns, leading to the increased release of low-molecular-weight compounds such as sugars and amino acids into the rhizosphere. These compounds can serve as readily available substrates for opportunistic pathogens, thereby promoting the growth and colonization of waterborne organisms such as Pythium spp (Sutton et al., 2006). Altered exudates can further enhance chemotactic attraction of motile zoospores toward root tissue, as amino acids including glutamic and aspartic acid released under stress conditions are known to elicit the complete pre-infection sequence of taxis, encystment, and germ-tube orientation at root surfaces, increasing the likelihood of infection (Donaldson and Deacon, 1993; Camli-Saunders and Villouta, 2025).

The agronomic significance of these physiological mechanisms is directly demonstrable in strawberries. Supplemental oxygenation of the fertigation solution significantly increased fruit yield from 42.48 g per plant under standard conditions to 58.98 g per plant, a 39% increase attributable solely to improved root-zone oxygenation, without negatively affecting fruit quality, establishing unambiguously that oxygen availability operates as a yield-limiting factor under typical fertigation regimes (Palencia et al., 2021). This evidence positions root-zone oxygen management not as an ancillary concern but as a primary determinant of productivity in soilless strawberry systems reliant on passive film aeration and continuous solution flow as the principal mechanisms of dissolved oxygen delivery. The cellular-level vulnerability described in this section, specifically the ATP depletion, membrane lipid disorganization, and altered root exudate profiles that develop under hypoxia, provides the mechanistic basis for the compounded *Pythium* susceptibility examined in section 3.4 of this review, and illustrates why oxygen deficit and pathogen pressure function as interacting rather than independent constraints in strawberry NFT systems.

### 3.4 NFT structure

The foundational design logic of NFT systems was codified by Cooper (1979) and reviewed by Graves (1983), who established that adequate root-zone supply of water, oxygen, and nutrients depends on three interacting hydraulic parameters: channel slope, flow rate, and channel length. In standard configurations, a channel slope of 1% is recommended to permit gravity-driven film flow while maintaining sufficient surface contact time; in practice, slopes of approximately 2.5 to 3.3% are more commonly implemented to prevent localized ponding caused by structural imprecision in the channel base (Cooper, 1979). Flow rates of approximately 1 L min^-^¹ per channel represent the operational standard, with a recommended maximum of 2 L min^-^¹, beyond which nutritional problems associated with excessive velocity have been documented; channel lengths are generally limited to 10–15 m to prevent nutrient and oxygen depletion at distal positions (Cooper, 1979). For strawberry specifically, Caruso et al. (2003) describe an NFT configuration consisting of rigid PVC gullies 12 cm wide, 10 cm deep, and 300 cm long, each supplied by a dedicated recirculating loop of a 220 L nutrient tank and a 90 W submersible pump, with channels arranged in paired vertical tiers at 70 cm and 120 cm above ground level. Channels wider than those used for leafy crops are specifically recommended for strawberry to accommodate root mat expansion over a multi-month production cycle (Burrage, 1992; Palmitessa et al., 2024).

The oxygen delivery mechanism in NFT is structurally distinct from substrate-based and deep-water culture systems: dissolved oxygen enters the nutrient solution exclusively through passive diffusion across the air-water interface of the thin film surface. This process is governed by the same mass transfer physics that underpin industrial gas absorption: according to penetration theory, the liquid-side mass transfer coefficient (k_L) is a function of the molecular diffusivity of oxygen in water and the contact time available at the gas-liquid interface (Higbie, 1935), while surface renewal theory further establishes that k_L is proportional to the square root of the rate at which fresh, oxygen-depleted solution is brought into contact with the air (Danckwerts, 1951). The volumetric oxygen transfer rate in the NFT channel is therefore the product of k_L and the specific interfacial area (a) available for gas exchange, a quantity directly determined by channel geometry and the extent of root mat coverage of the film surface (Cooper, 1979; Palmitessa et al., 2024). Empirical evidence confirms this dependency: Urrestarazu et al. (2005) recorded dissolved oxygen declining from 6.2 to 2.9 mg/L along a conventional NFT channel (**Figure 2B**), and López-Pozos et al. (2011) demonstrated that increasing channel slope from 2% to 4% measurably reduced dissolved oxygen depletion and improved tomato yield, consistent with the prediction that steeper slopes increase surface renewal rate and thereby raise k_L. In short-cycle crops with small, stable root systems, this mechanism is adequate under thermally stable conditions: the film remains shallow, the air-water interface is maximized, and residence time is short. In strawberry, however, root biomass accumulates progressively over a production cycle of six months or more, physically reducing the effective cross-sectional channel area available for film flow, increasing local hydraulic resistance, and critically submerging portions of the root mat that would otherwise be exposed to air (Burrage, 1992; Palmitessa et al., 2024). This progressive occlusion reduces the specific interfacial area (a) and disrupts surface renewal precisely as root respiratory demand is rising toward peak fruit development (**Figure 2A**), creating a structural deterioration in oxygen transfer capacity that is inherent to the geometry of the system and cannot be corrected by adjusting slope or flow rate alone. Because NFT channels provide no substrate matrix to buffer solution chemistry or retain oxygen between flow cycles, any interruption to hydraulic continuity, whether from pump failure, channel blockage, or root mat-induced flow stagnation, eliminates the sole oxygen delivery pathway to the root zone entirely (Burrage, 1992; Jackson et al., 1984).

The mechanistic relationship between channel geometry, interfacial area, and oxygen transfer rate suggests that gutter design itself represents an underexplored variable in NFT optimization for strawberry, a crop whose root system is markedly different from the leafy crops for which most commercial NFT hardware was originally designed. Unlike lettuce or spinach, strawberry develops a dense, fibrous root mat that expands progressively over six months or more, simultaneously degrading both k_L and a; designing channel geometry around this specific root development trajectory, rather than adapting short-cycle crop infrastructure to a long-cycle species, may represent a more effective path toward maintaining adequate dissolved oxygen delivery during the critical fruiting period.

### 3.5 Oxygenation interventions and their limitations

Given the structural inadequacy of passive film aeration for long-cycle strawberry production, the literature describes several classes of intervention aimed at supplementing or restoring dissolved oxygen delivery. These can be grouped into three categories: direct solution oxygenation, hydraulic and system design modification, and temperature management. Each addresses a distinct component of the oxygen deficit problem, but none has been validated across a full six-month strawberry production cycle under commercial conditions, and the interactive efficacy of combining them remains uncharacterized.

Direct solution oxygenation is the most empirically supported intervention. Palencia et al. (2021) demonstrated that oxyfertigation, the injection of oxygen-enriched water into the fertigation stream, increased strawberry fruit yield by 39% relative to standard fertigation in soilless production, the most direct quantitative evidence that solution-borne oxygen is yield-limiting under typical practice. Jackson et al. (1984) documented that forced supplemental aeration introduced into operating NFT channels measurably improved dissolved oxygen concentrations, establishing that passive film diffusion alone is insufficient under the flow and root-density conditions that develop across a crop cycle. Suhl et al. (2019) characterized oxygen consumption dynamics in recirculating NFT, confirming that biological oxygen demand accumulates progressively with root biomass and microbial load, providing the mechanistic rationale for continuous rather than intermittent supplemental oxygenation in long-cycle systems. Recent studies indicates that oxygenation interventions, particularly aeration, can introduce unintended effects on root-zone processes, including disruption of the rhizosphere and reduced nutrient uptake under certain conditions (Langenfeld and Bugbee, 2025), while broader analyses of NFT systems continue to emphasize persistent challenges associated with oxygen management and system design (Palmitessa et al., 2024). Beyond conventional aeration, emerging technologies including micro-nanobubble oxygen injection into nutrient solutions have demonstrated significant improvements in root development, photosynthetic rate, and yield in soilless cultivation systems (Wu et al., 2019; Zhao et al., 2024), though their efficacy and cost profile under the specific hydraulic and biological oxygen demand conditions of long-cycle strawberry NFT remain uncharacterized. The operational constraints of continuous direct oxygenation, equipment cost, pressure management in recirculating systems, and the risk of selectively promoting aerobic microbial communities that further increase biological oxygen demand, have not been systematically evaluated under commercial strawberry NFT conditions.

Hydraulic and system design interventions address the structural component of oxygen transfer deterioration. López-Pozos et al. (2011) demonstrated that increasing channel slope from 2% to 4% measurably reduced dissolved oxygen depletion and improved yield in tomato NFT, consistent with surface renewal theory predicting that steeper slopes increase the liquid-side mass transfer coefficient. Restricting channel length to below 10–15 m limits the severity of longitudinal DO gradients at distal root positions, a constraint codified in the original NFT design parameters by Cooper (1979) and Graves (1983). Wider channels, specifically recommended for strawberry to accommodate root mat expansion by Burrage (1992) and Palmitessa et al. (2024), reduce the proportional impact of root occlusion on film cross-section and gas-liquid interfacial area. The fundamental limitation of all hydraulic interventions is that they optimize k_2_ and interfacial area at system installation but cannot compensate for the progressive root mat development that degrades both parameters across a six-month strawberry cycle: channel slope and dimensions can be selected at planting, but expanding root biomass will progressively override these design choices regardless, particularly during the peak fruiting period when respiratory demand is highest.

Temperature management constitutes the third category and uniquely among interventions addresses both the oxygen solubility component of DO deficit and the *Pythium* susceptibility compound simultaneously. Reducing root-zone solution temperature from 25 °C toward the optimal range of 18–22 °C increases oxygen solubility in the nutrient solution by approximately 15–20% (dependent on solute concentration), reduces root respiratory demand, maintains the root-zone thermal stability required for normal strawberry physiology, and suppresses the temperature-triggered necrotrophic transition in *Pythium*-coloni (Gonzalez-Fuentes et al., 2016; Sakamoto et al., 2016; Hooks et al., 2022; Sutton et al., 2006). In commercial greenhouse and vertical farm environments where ambient air temperatures rise substantially in summer, active chilling of the recirculating solution represents the most integrated management response available, addressing dissolved oxygen limitation and pathogen pressure through a single intervention, but the capital and operating costs of solution chilling over a multi-month production cycle represent a significant economic barrier that has not been formally evaluated against the yield and loss costs of unmanaged summer oxygen depletion in strawberry NFT.

In aggregate, the three intervention categories address different components of the oxygen deficit chain, and the available evidence indicates that no single intervention is sufficient for a six-month strawberry cycle under warm commercial production conditions. The most plausible path toward adequate oxygenation involves combining direct solution oxygenation with temperature management and channel design optimization selected for the specific root development trajectory of strawberry rather than adapted from short-cycle crop infrastructure. The cost, operational complexity, and interactive efficacy of such a combination over a full strawberry production cycle have not been evaluated, and this absence of system-level intervention evidence connects directly to the structural and threshold characterization gaps identified in the following section.

### 3.6 NFT gaps and limitations

The evidence reviewed above establishes a mechanistic chain from channel geometry to root-zone physiology: oxygen delivery in NFT depends on k_L × a, both components of which are degraded by progressive root mat development, precisely during the period of highest respiratory demand in strawberry. Yet this chain, while mechanistically coherent, is not empirically closed. No published study has characterized how increasing root occupancy in strawberries modifies film thickness, effective hydraulic radius, or longitudinal dissolved oxygen gradients within operating channels across a full production cycle. The 40% seasonal decline in dissolved oxygen documented by Palencia et al. (2021) and the channel-length depletion gradients recorded by Urrestarazu et al. (2005) and Gislerød and Kempton (1983) confirm that these dynamics are agronomically real (**Figure 2C**), but neither study isolated the structural contribution of root mat development from the concurrent effects of rising solution temperature and increasing crop respiratory demand. This distinction matters: if root mat occlusion is the dominant driver of interfacial area loss, then hydraulic interventions such as slope modification are limited in their corrective capacity, as López-Pozos et al. (2011) demonstrated for tomato, but which has not been tested in strawberry across geometries relevant to commercial production. The absence of this structural characterization means that the interaction between channel geometry and oxygen transfer performance over a six-month strawberry crop cycle remains entirely unmodelled.

This hydraulic gap connects directly to a second, more fundamental problem: the absence of a strawberry-specific dissolved oxygen threshold. Every quantitative threshold currently available derives from other species. Gislerød and Kempton (1983) observed growth suppression at 1–3 mg/L in cucumber and tomato; Zheng et al. (2007) documented progressive physiological decline in tomato across sub-saturating concentrations. Applying these values to strawberry management implicitly assumes cross-species physiological equivalence that the evidence reviewed here does not support. Strawberry possesses no documented capacity for adaptive aerenchyma formation, its entire vascular connection to the shoot passes through a compact crown with no internal oxygen buffering, and its roots exhibit rapid ATP depletion and fermentative metabolism onset under even moderate hypoxia, characteristics that distinguish it physiologically from both tomato and cucumber. Whether the critical threshold in strawberry lies above or below the 1–3 mg/L range established for other crops is unknown, and that uncertainty has direct consequences for any oxygen management protocol applied to NFT strawberry production.

What this means practically is that two things need to happen in sequence before NFT strawberry systems can be rationally designed around oxygen management. First, the relationship between root mat development and hydraulic performance must be characterized within channel geometries spanning the range used in commercial strawberry production, specifically, how effective cross-sectional flow area, film thickness, and longitudinal DO gradients change between transplant and peak fruiting in channels of the widths and lengths currently deployed. Second, and dependent on that characterization, a dose-response study exposing strawberries to a range of controlled dissolved oxygen concentrations under NFT-relevant flow and temperature conditions is needed to establish the physiological threshold at which nutrient uptake, root respiration, and marketable yield are measurably impaired. Until both are available, oxygen management in strawberry NFT will remain based on thresholds and geometries inherited from crops with fundamentally different root architecture and oxygen tolerance, an inadequate foundation for a production system where oxygen delivery failure is both structurally predictable and agronomically consequential.

## 4 *Pythium* root rot in NFT strawberry systems

### 4.1 Introduction and scope

Strawberries in NFT occupy a structurally unique position in the *Pythium* pathosystem, one that cannot be fully understood by extrapolation from the extensive literature on other hydroponic crops. Three compounding species-specific vulnerabilities converge in this crop-system combination: the exceptionally high root-zone oxygen demand of *Fragaria* relative to other hydroponic crops makes this species disproportionately sensitive to the oxygen depletion that accelerates through dense NFT root mats; the primary *Pythium* species documented in strawberry NFT (*P. dissotocum*) exhibits a colonization strategy that selectively targets the stele of immature root tips, inflicting subclinical damage that suppresses yield before above-ground symptoms are detectable; and the six-month production cycle of commercial strawberry NFT generates chronic inoculum accumulation pressure that is categorically different from the short cycles for which most hydroponic epidemiological data were produced. The consequence is that quantitative disease management thresholds, management efficacy data, and cultivar susceptibility information derived from tomato, spinach, lettuce, and pepper hydroponics, the crops from which the overwhelming majority of published *Pythium* data originate, cannot be applied to strawberry NFT without explicit and largely unwarranted assumptions about biological equivalence. Naming this data gap is the most important contribution this section can make.

Within recirculating hydroponic systems broadly, zoosporic pathogens represent the defining disease problem: Stanghellini and Rasmussen (1994) documented 21 fungal, four viral, and three bacterial pathogens capable of causing root disease in hydroponic crops, with zoosporic fungi accounting for the most extensive crop losses, and identified solution temperature as the primary governor of disease severity, a finding that connects directly to NFT’s limited thermal buffering capacity. *Pythium* is the primary biotic threat in long-cycle NFT culture due to its capacity to exploit the structural vulnerabilities inherent to recirculating media-free systems, particularly continuous nutrient solution recirculation, dense uniform host plantings, and the oxygen dynamics that NFT channels generate (Paludan, 1985; Paulitz, 1997; Stanghellini and Rasmussen, 1994; Sutton et al., 2006; Zhang and Tu, 2000). Unlike field-grown crops, NFT systems lack the natural soil microbial diversity that suppresses pathogen establishment, and the recirculating nutrient solution provides a continuous dispersal pathway that amplifies any initial inoculum event into a system-wide outbreak with a speed and scale that has no equivalent in substrate-based production (Gravel et al., 2006; Koohakan, 2007; Sutton et al., 2006). The mechanistic relationship between dissolved oxygen deficiency and *Pythium* susceptibility, a relationship with particular significance for strawberries, as developed below, is examined in depth in the dissolved oxygen section of this review.

### 4.2 *Pythium* biology and species of concern

*Pythium* is a phytopathogenic oomycete genus of over 200 species whose water-borne zoospores are the primary agents of infection in recirculating hydroponic systems, sustaining motility continuously in the water-rich conditions of NFT culture (Sutton et al., 2006; Uzuhashi et al., 2010). Zoospores navigate aqueous environments chemotactically toward root exudates, particularly glutamic and aspartic acid, eliciting the complete pre-infection sequence of taxis, encystment, and germ-tube orientation at root surfaces (Donaldson and Deacon, 1993; Camli-Saunders and Villouta, 2025).

Strawberry presents a particularly vulnerable profile within this chemotactic framework. Fragaria × ananassa roots are classified as weak respires, characterized by low metabolic rates, sparse branching, and partial suberization that limits the absorptive surface available for oxygen uptake (Dickmann and Blanke, 2000). At 20 °C, measured root respiration in frigo-stage plants ranges from 0.70 to 0.73 mg CO2 g-1 fresh mass h-1, rising from near-zero at 1 °C and accumulating approximately 1.4–1.6 g CO2 per plant over the six-week developmental period preceding harvest (Dickmann and Blanke, 2000). This morphological and metabolic profile means that the hypoxic microenvironments that develop within NFT root mats as channel length increases, and which are characterized in detail in the dissolved oxygen section of this review, reach physiologically damaging oxygen concentrations for strawberry at threshold levels that the more robust root architectures of tomato or cucumber can better buffer. Oxygen-stressed roots exhibit altered exudate profiles, increasing the attractiveness of the rhizosphere to zoospore chemotaxis and further concentrating infection pressure at the root tips most susceptible to colonization (Camli-Saunders and Villouta, 2025; Cherif and Belanger, 1992; Donaldson and Deacon, 1993; Sutton et al., 2006).

In strawberry, *Pythium* species are consistently associated with root and crown rot, damping off, and associated wilting and stunted growth that cause substantial economic losses across developmental stages from seedling establishment through mature fruiting (Hendrix and Campbell, 1973; Sanogo and Moorman, 1993; van der Plaats-Niterink, 1981; Van West et al., 2003). In mature plants, infections induce necrotic lesions on root tips and feeder roots, directly disrupting mineral uptake and reducing marketable yield (Martin and Loper, 1999; Sanogo and Moorman, 1993). Critically, *Pythium* root rot is readily misdiagnosed in NFT systems because above-ground symptoms, wilting, chlorosis, and growth suppression, are frequently attributed to nutrient deficiency or dissolved oxygen stress before the pathogenic cause is identified (Mattson, 2018). Stanghellini and Kronland (1986) demonstrated in lettuce hydroponics that subclinical infection of feeder rootlets by *P. dissotocum* reduced yield measurably without producing visible crown rot symptoms, a finding with direct implications for strawberry NFT, where extensive subsurface colonization may be well advanced before growers are alerted by shoot-level indicators.

The host range of *Pythium* varies substantially across species. *Pythium ultimum* infects over 300 host species, while others such as *P. uncinulatum* are restricted to a single host (Farr et al., n.d.). In hydroponic systems, the predominant species are *P. aphanidermatum, P. irregulare, P. ultimum* var. *ultimum*, and *P. dissotocum*, with *P. dissotocum* notably colonizing the stele of immature root tips in strawberry, a more aggressive colonization pattern than the cortical infection typical of most other crop hosts, with direct consequences for vascular function and resource uptake (Sutton et al., 2006). In California strawberry production fields, *P. ultimum* is the most frequently recovered species alongside *P. irregulare* and *P. paroecandrum* (Martin, 1999), while field studies in Argentina have reported *P. aphanidermatum*, *P. spinosum*, and *P. ultimum* (J.M. Ibañez et al., 2022). Species pathogenicity toward strawberry varies considerably, complicating management strategies that rely on species-level diagnosis, and no systematic survey of species distribution in strawberry NFT systems under commercial production conditions has been published (Broders et al., 2007; Dorrance et al., 2004; Harvey et al., 2001) (**Table 1**).

**Table 1:** *Pythium* spp. and related oomycetes of relevance to strawberry NFT production: temperature optima, dissolved oxygen sensitivity, root infection site, and strawberry host documentation.

Species	Temperature (min/opt/max)	DO sensitivity/relationship	Strawberry pathogenicity	Key references
Group A: Species with confirmed or probable pathogenic relevance to strawberry and/or hydroponic systems				
*Pythium ultimum* Trow	Min ~0 °C; opt. 20–22 °C; max 35–36 °C; no growth at 38–40 °C	Low DO predisposes to infection; not pathogenic in soilless culture regardless of DO regime	Pathogenic in peat-sand; not pathogenic in rockwool or nutrient solution; rarely produces zoospores (limited waterborne dispersal)	(van der Plaats-Niterink, 1981) (Moulin et al., 1994) (Sutton et al., 2006) (Pánek and Střížková, 2021)
*Pythium aphanidermatum* (Edson) Fitzp.	Min 10 °C; opt. 28–35 °C; max >40 °C; symptomless colonization at 16–18 °C → acute disease within hours upon warming to 24–28 °C	Low DO predisposes roots; temp–DO coupling critical: higher temps reduce DO solubility and accelerate pathogen growth simultaneously	Confirmed pathogenic in true soilless culture; virulent on 5 strawberry cvs.; isolated from Czech and Argentinian strawberry farms	(van der Plaats-Niterink, 1981) (Moulin et al., 1994) (Owen-Going et al., 2003) (Sutton et al., 2006) (Ibañez et al., 2022)
*Pythium dissotocum* Drechs.	Opt. ~25 °C (range 5–35 °C); displaced by P. aphanidermatum above 23 °C; dominant in cool-season production (<23 °C)	Most direct DO evidence of any Pythium sp.: moderate O_2_ (2.5–3 ppm) yielded greater colonization than low O_2_; suppressed at high O_2_ (5–7 ppm)	Confirmed pathogenic in strawberry (stelar invasion, Nemec, 1972); 11% of isolates from Czech strawberry farms; component of black root rot complex	(Nemec, 1972)eme (Stanghellini and Kronland, 1986) (Chérif et al., 1997) (Sutton et al., 2006) (Pánek and Střížková, 2021)
*Pythium irregulare* Buisman	Min 1–4 °C; opt. 27–30 °C; max 35–37 °C; greater damping-off at 12 °C than 25 °C; wide intraspecific variation	Not pathogenic in soilless culture regardless of DO regime	Pathogenic in peat-sand; not pathogenic in rockwool or nutrient solution; confirmed in strawberry in Argentina (Koch’s postulates fulfilled)	(van der Plaats-Niterink, 1981) (Moulin et al., 1994) (Sutton et al., 2006) (Barr et al., 1997) (Ibañez et al., 2022)
*Pythium sylvaticum* W.A. Campbell & F.F. Hendrix	Opt. 18–23 °C; greater pathogenicity at 18 °C than 13 °C; not pathogenic in soilless systems	Not pathogenic in soilless systems regardless of DO regime	Confirmed in strawberry (Koch’s postulates); first Americas record on cv. Chandler; part of strawberry BRR complex in Japan	(van der Plaats-Niterink, 1981) (Moulin et al., 1994) (Shrestha et al., 2023) (Watanabe et al., 1977)
Group B: Additional oomycete species with strong hydroponic or strawberry relevance				
*Phytopythium helicoides* (Drechs.) Abad et al.	Thermophilic; opt. 35–40 °C; max 43 °C; warm-season pathogen	No species-specific DO data available	Significant strawberry pathogen across multiple countries; up to 50% plant mortality on cv. Florida Radiance; confirmed in Japan, Florida, and Argentina	(Ishiguro et al., 2014) (Marin et al., 2019) (Ibañez et al., 2022)
*Pythium myriotylum* Drechs.	Tropical/subtropical; warm-season pathogen; no cardinal temperatures in accessible sources	No species-specific DO data available	Primary pathogen in strawberry stunt disease complex in Japan; caused epidemic killing ~50% of 30,000 hydroponic lettuce plants (US Virgin Islands)	(Watanabe et al., 1977) (Stanghellini et al., 1998) (Amrhein et al., 2025)

Species are organized into Group A (five *Pythium* spp. with confirmed or probable pathogenic relevance to strawberry and/or hydroponic systems) and Group B (two additional oomycetes with strong hydroponic or strawberry relevance in recent surveys). Stelar invasion denote the depth of tissue penetration documented by histopathology in *Fragaria* × *ananassa* roots (Nemec, 1972). ‘not pathogenic in soilless culture’ refers to the finding of Moulin et al. (1994) that *P. irregulare*, *P. sylvaticum*, and *P. ultimum* caused disease in peat-sand substrate but not in rockwool or nutrient solution. Superscript numbers identify the source of each specific claim; full citations are provided in the reference list below. DO, dissolved oxygen; NFT, nutrient film technique; BRR, black root rot.

### 4.3 Epidemiology in recirculating NFT systems

*Pythium* inoculum enters NFT systems through airborne dust, contaminated water, plant debris on tools and footwear, or exposed transplants from plug and bare-root production systems (Hong and Moorman, 2005; Moorman et al., 2002; Paulitz, 1997; Sutton et al., 2006). Once introduced, proliferation in recirculating nutrient solutions can be extremely rapid. In hydroponic spinach, *P. aphanidermatum* caused complete shoot and root loss within three to four days of inoculation, while *P. dissotocum* caused severe damage, reducing shoot biomass by 74% and root biomass by 83% (Gold and Stanghellini, 1985). In tomato hydroponics, initial inoculum levels as low as 6.7 CFU/mL were sufficient to initiate infection, with populations reaching 1,030 CFU/mL in untreated systems after four weeks (Zhang and Tu, 2000). Sutton et al. (2006) found that all isolates of *P. aphanidermatum* and *P. dissotocum* tested in sweet pepper single-plant hydroponic units rapidly colonized root systems within days of zoospore inoculation at 5 × 10³ zoospores/mL, producing architecture changes, root-tip browning, and growth suppression across all experimental repetitions, with disease severity amplified by higher temperature and light conditions.

It is important to state explicitly what the preceding data do and do not indicate. Every quantitative epidemiological measurement cited above (inoculum thresholds, NFT gutters and reservoirs are particularly vulnerable because zoospores travel freely in the nutrient solution; the infrastructure itself, including pipes, tanks, and gutter surfaces, functions as a persistent inoculum reservoir between crop cycles, enabling rapid system-wide colonization from a single infection point (Holderness and Pegg, 1986; Pettitt et al., 1998; Postma et al., 2008; Stanghellini and Kronland, 1986; Sutton et al., 2006). Stagnant zones within channels arising from root mat development further promote pathogen persistence between sanitation events (Sutton et al., 2006). In CEA environments, insect vectors including fungus gnats (*Bradysia* spp.) and shore flies (*Scatella stagnalis*) further facilitate dispersal of *Pythium* propagules between plants (Braun et al., 2012).

### 4.4 The dissolved oxygen–*Pythium* nexus in strawberry NFT

The dissolved oxygen–*Pythium* relationship in NFT strawberry deserves explicit treatment here rather than wholesale deferral to the dissolved oxygen section of this review, because it constitutes the central mechanistic argument linking NFT’s structural design to strawberry’s disease vulnerability. NFT channels generate a characteristic dissolved oxygen gradient: the solution enters the proximal end of the channel near atmospheric saturation, and oxygen is consumed progressively along the channel as it supports root respiration, microbial metabolism in the biofilm, and degradation of organic exudates (Graves, 1983; Sutton et al., 2006). The rate of oxygen depletion accelerates non-linearly as root mat density increases across a long crop cycle, with the distal sections of channels in commercial installations commonly falling below phytotoxic thresholds before the growing season is complete (Dorais et al., 2008; Graves, 1983).

For strawberries, this gradient is more physiologically consequential than for most other hydroponic crops, due to this species’ elevated root oxygen demand. Cherif and Belanger (1992) found that *Pythium* colonization of hydroponically grown tomato roots was significantly greater under moderate (5.8–7.0%) and low (0.8–1.5%) oxygen conditions than at higher concentrations (11–14%), with oxygen stress increasing lipoxygenase activity and the consequent disorganization of membrane lipids providing the biochemical basis for enhanced pathogen colonization. While this foundational work was conducted in tomato, the implications for strawberry are more severe: given strawberry’s higher root oxygen demand, the tissue-level hypoxia that activates this lipoperoxidation cascade will be reached at higher absolute dissolved oxygen concentrations in solution than in tomato, meaning that NFT oxygen levels that are sub-optimal but non-damaging for tomato may already constitute active physiological stress for strawberry roots, creating the membrane-level vulnerability that *Pythium* requires to shift from biotrophic to necrotrophic infection.

Temperature compounds this interaction by affecting both the severity of the biotrophic-to-necrotrophic transition and the rate of oxygen depletion in solution. Owen-Going et al., (2003) observed that roots of hydroponic pepper colonized by *P. aphanidermatum* or *P. dissotocum* at 16–18 °C can remain symptomless, yet develop severe visible decay within hours when temperature rises to 24–28 °C, a biotrophic latency that has direct relevance to the seasonal temperature fluctuations experienced in commercial NFT operations. Since oxygen solubility in water decreases as temperature rises, the periods of highest summer temperature simultaneously reduce ambient oxygen availability, accelerate root respiration, and trigger the necrotrophic phase in already-colonized roots, a convergence of stressors that can manifest as catastrophic crop loss from a single thermal event (Owen-Going et al., 2003; Sutton et al., 2006). Whether this temperature-triggered disease cascade operates in strawberry NFT with the same kinetics as demonstrated in pepper remains unestablished.

### 4.5 Environmental and system drivers

Beyond dissolved oxygen and temperature, *Pythium* infections in NFT systems are modulated by solution pH, moisture dynamics, and the organic composition of the root-zone environment (Lifshitz and Hancock, 1984; Sutton et al., 2006). Among these, temperature is the most consistently documented driver: solution temperatures above 25 °C favor pathogen growth, exacerbate host stress, and increase disease severity, with *P. aphanidermatum* and *P. dissotocum* exhibiting peak virulence above 30 °C, while cooler conditions tend to favor less aggressive species such as *P. ultimum* (Bates and Stanghellini, 1984; Gold and Stanghellini, 1985; Miyake et al., 2014; Owen-Going et al., 2003) (**Table 1**). pH plays a complementary role: values below 5.0 have been associated with reduced oomycete disease incidence, while elevated pH increases plant susceptibility, and the formation of appressoria varies with both temperature and pH (Endo and Colt, 1974; Gillespie, 2019).

NFT-specific system parameters that receive insufficient attention in the *Pythium* literature include channel length, flow rate, and film depth, each of which directly determines the oxygen gradient profile and the residence time of zoospores in solution. Longer channels create steeper oxygen depletion gradients, increasing the proportion of root surface area exposed to hypoxic conditions; lower flow rates reduce turbulent reaeration at the film surface; and shallow film depth amplifies the proportional impact of root mat obstruction on solution velocity and gas exchange. These design parameters interact with pathogen dispersal dynamics: zoospores carried in the solution film will be deposited preferentially in the low-velocity zones that develop behind dense root obstructions, concentrating infection pressure at the exact sites of greatest oxygen stress. No systematic study has characterized these design-disease relationships in strawberry NFT under conditions representative of commercial production. Excess moisture, poor ventilation, and accumulation of decomposing organic matter in NFT gutters further sustain pathogen populations under conditions of low microbial competition).

### 4.6 Management strategies

Effective management of *Pythium* in hydroponic strawberry requires a critical distinction that the literature often obscures there is a fundamental difference between treating the bulk recirculating solution and protecting the root zone itself, and many commonly recommended technologies operate effectively only in the former context while failing in the latter, which is where most *Pythium* infection and colonization actually occur.

Environmental control remains the first-line defense. Maintaining nutrient solution temperatures below 25 °C and sustaining dissolved oxygen above minimum thresholds are the two most critical controls for limiting infection risk, and in strawberry NFT, where both parameters interact to compound disease susceptibility as documented above, these are not optional management practices but prerequisites for system viability (Bates and Stanghellini, 1984; Gold and Stanghellini, 1985). Routine flushing cycles, smooth-walled NFT gutters, and consistent disinfection of tools and reservoirs reduce inoculum persistence (Sutton et al., 2006).

Water treatment technologies including ultraviolet irradiation, ozonation, and filtration provide pathogen suppression within recirculating solutions but face a critical structural limitation in NFT systems: the majority of *Pythium* zoospores that cause infection are dispersed locally among roots within the root zone, and those that enter the bulk recirculating solution must survive turbulent passage through pipes and mixing tanks to reach the UV unit, a sequence from which few propagules emerge viable (Sutton et al., 2006). Consistent with this, UV treatment at doses sufficient to eliminate *Pythium* propagules provided little or no suppression of root rot in lettuce and chrysanthemum through experiments and failed to significantly suppress root rot increase in a commercial-scale pepper NFT crop (Sutton et al., 2006). This is a critical negative finding that should govern how UV is positioned in integrated management programs for strawberry NFT: UV can reduce the inoculum load in solution between crop cycles and prevent long-distance dispersal events, but it is not an effective root-zone protectant in a live crop. Ozonation provided broad-spectrum disinfection in rockwool-based systems (Runia, 1994), and filtration using a 7 µm cartridge effectively removed *Pythium* zoospores from cucumber nutrient solutions (Goldberg et al., 1992), but efficacy of these approaches for root-zone protection in strawberry NFT remains undemonstrated.

Biosurfactant-based approaches represent a further management option with biological plausibility under NFT conditions: rhamnolipid biosurfactants produced by Pseudomonas spp. rapidly lyse *Pythium* zoospore plasma membranes at concentrations between 25 and 60 µg/mL, eliminating motility and viability within seconds of exposure (Stanghellini and Miller, 1997). Because this mode of action targets the zoospore directly in the aqueous environment, it avoids the root-zone localization problem that limits UV efficacy and could theoretically provide continuous zoospore inactivation within the recirculating film. Integration of this approach into commercial strawberry NFT management protocols has not been reported. Silicon amendments to recirculating nutrient solutions at 1.7 mM have suppressed *Pythium* root rot in cucumber hydroponics through induction of plant defense responses (Cherif and Belanger, 1992); whether this approach translates to strawberry, a weak silicon accumulator, warrants investigation as a low-input management adjuvant.

Biological control agents including Trichoderma spp., endophytic actinobacteria, and plant growth-promoting rhizobacteria can suppress *Pythium* through competition, antibiosis, and induction of host defenses (Misk and Franco, 2011; Wu et al., 2020). Several Pseudomonas strains, including *P. corrugata* 13 and *P. aureofaciens* 63-28, suppressed *P. aphanidermatum* root rot in cucumber by inducing salicylic acid and activating defense enzymes including phenylalanine ammonia-lyase and peroxidase (Chen et al., 2000, 1999). The persistence of biocontrol agents under commercial conditions is the central practical challenge: Pseudomonas chlororaphis Tx-1 maintained stable populations in sweet pepper roots inoculated with *Pythium* but declined in non-inoculated plants (Chatterton et al., 2004), and the population densities required for sustained suppressive activity remain poorly characterized under strawberry NFT hydraulics over a six-month cycle (Paulitz, 1997; Postma et al., 2008). Chemical fungicides including metalaxyl, azoxystrobin, fosetyl-Al, and pyraclostrobin remain effective against *Pythium* spp., though resistance management must be incorporated to preserve long-term efficacy in long-cycle systems (Lookabaugh et al., 2015; Stiles et al., 2005; Taylor et al., 2002). While no strawberry cultivar with complete *Pythium* resistance has been identified, breeding efforts to characterize tolerance-associated traits continue (Lookabaugh et al., 2015; Pánek and Střížková, 2021; Stiles et al., 2005).

In aggregate, the current evidence base supports multi-barrier preventive management centered on the root zone, temperature control, dissolved oxygen maintenance, and biological root-zone colonization by beneficial microorganisms, rather than bulk-solution disinfection as the primary containment strategy for *Pythium* in strawberry NFT. This reorientation is important because most published management recommendations emphasize water treatment technologies, which are more straightforward to retrofit into commercial systems, while the root-zone colonization approaches that the mechanism argues are more directly protective remain less commercially developed.

### 4.7 Research gaps and future directions

The most important research gap identified by this review is the absence, in any published form, of epidemiological data specific to the strawberry-*Pythium*-NFT pathosystem. Every quantitative parameter that would be required to design evidence-based disease management for this system, inoculum threshold levels, epidemic development rates, yield-loss functions, biocontrol population persistence thresholds, and combined DO-temperature disease prediction models, has been measured in other crops (principally tomato, pepper, spinach, and lettuce) in other system configurations (principally short-cycle deep water or rockwool culture). The biological and system differences between these source contexts and strawberry NFT are substantial enough that applying these data to strawberry NFT management constitutes an assumption with unknown error. Establishing this dedicated experimental base is the prerequisite for rational, evidence-driven management of the disease in commercial systems.

Within this overarching gap, several specific research priorities can be identified. First, the inoculum thresholds and epidemic dynamics specific to strawberries in NFT under commercial flow and temperature conditions need to be characterized quantitatively, for the species, particularly *P. dissotocum*, that colonize strawberry stele rather than cortex. Second, the interaction between NFT channel design parameters (length, flow rate, film depth) and *Pythium* infection risk in strawberries needs to be studied as a system: channel geometry determines the oxygen gradient and zoospore deposition patterns, both of which are central to disease pressure, and neither has been experimentally optimized for strawberries in NFT. Third, the DO-temperature convergence identified in the environmental drivers section, where summer temperature simultaneously reduces oxygen availability, triggers necrotrophic transitions in colonized roots, and accelerates pathogen growth, should be characterized in strawberry-specific bioassays to establish predictive thresholds that could support early warning protocols.

Molecular and genomic tools offer a further avenue for accelerating progress in cultivar-level management. While marker-assisted selection and QTL analysis have enabled identification and introgression of resistance loci in major field crops, equivalent phenotyping platforms and genomic resources for *Pythium* tolerance in strawberry have not been systematically developed (Karthikeyan et al., 2022; Klepadlo et al., 2019; Whittington et al., 2014). Developing strawberry-specific resistance screening protocols under NFT-relevant inoculation conditions (replicating the DO gradient, temperature range, and *P. dissotocum* species) would accelerate breeding progress and produce tolerance data that is directly translatable to commercial system design.

At the system level, cost-benefit analyses of integrated multi-barrier approaches are needed to evaluate economic feasibility under commercial strawberry NFT conditions specifically. Multi-barrier frameworks incorporating UV irradiation, slow-sand biofiltration, biological control agents, and optimized channel design have demonstrated collective effectiveness in recirculating systems (Ehret et al., 2001; Pettitt et al., 1998; Runia, 1994), but the financial and operational trade-offs of combining these approaches over a six-month strawberry production cycle have not been formally evaluated. Slow-sand biofiltration in particular represents an underexplored option, offering simultaneous pathogen removal and microbial antagonist cultivation at lower energy cost than UV or ozone systems (Ehret et al., 2001). Emerging evidence on microbiome management in recirculating systems further supports system-level prevention frameworks rather than single-barrier approaches (Chatterton et al., 2004; Stouvenakers et al., 2020; Thomas et al., 2024). Until the strawberry-*Pythium*-NFT pathosystem has its own empirical epidemiological foundation, however, all management recommendations for this system, including those offered in this review, rest on inference from biologically dissimilar hosts and system configurations, a limitation that should drive concerted experimental investment in this understudied pathosystem.

## 5 Discussion

The mechanistic literature reviewed in the preceding sections converges on a conclusion that it is not self-evident from reading either the dissolved oxygen or the *Pythium* literature in isolation: the two primary failure modes of NFT strawberry are not parallel technical problems requiring separate solutions, but coupled failure modes sharing a common mechanistic pathway through root-zone oxygen status (**Figure 1**). This distinction matters for how research priorities are set and how management strategies are evaluated. A system designed to supply adequate dissolved oxygen throughout a six-month fruiting cycle under commercially relevant temperature conditions would simultaneously reduce the hypoxia-driven membrane deterioration that predisposes *Fragaria* × *ananassa* roots to colonization, suppress the biotrophic-to-necrotrophic transition in already-colonized tissue, and diminish the rhizosphere exudate profile that drives chemotactic zoospore accumulation at the root surface. Conversely, a system that fails on oxygen delivery does not simply incur a yield penalty from hypoxia; it concurrently elevates disease vulnerability in a host that, lacking documented aerenchyma formation capacity and relying entirely on external solution oxygen supply, has no internal buffer against that failure. This synergy means that the agronomic risk of NFT strawberry is non-linear: small deficits in oxygen management translate into disproportionately large disease risk, particularly for thermophilic *Pythium* species whose infection efficiency peaks under the same warm conditions that drive oxygen depletion.

What separates the current state of knowledge from a foundation adequate for rational system design is not primarily the absence of mechanistic understanding but the absence of quantitative parameters specific to this crop-system-pathogen combination. The physics of gas-liquid mass transfer, the biology of zoospore dispersal, and the cellular consequences of root hypoxia are all well-characterized. What does not exist is the quantitative closure of those frameworks under strawberry NFT conditions: how root mat development modifies hydraulic performance in commercially deployed channel geometries; what dissolved oxygen concentration constitutes a physiologically meaningful threshold for yield loss in *Fragaria* × *ananassa* specifically; and what inoculum densities, epidemic development rates, and host defense competence profiles define the disease envelope for the strawberry-*Pythium*-NFT pathosystem. These gaps, identified independently in Sections 3.7 and 4.6, are not separable: the dissolved oxygen threshold study cannot be meaningfully designed without the hydraulic characterization, and the *Pythium* management framework cannot be rationally calibrated without knowing the oxygen regime that strawberry roots actually experience in production-scale channels. The research sequence is therefore fixed by the logic of the system, not by investigator preference.

The strategic case for resolving these constraints has strengthened materially since NFT was abandoned in the 1980s. At that time, the failure of a substrate-free system could be absorbed because peat- and coir-based alternatives were agronomically adequate and environmentally uncontroversial. Neither condition holds today. The life cycle liabilities of substrate-based production, documented through peat carbon accounting and coir transport emissions analyses (Stichnothe, 2022; Woznicki et al., 2024), are now visible to regulators and supply chain actors in ways that create genuine commercial pressure for substrate elimination. A substrate-free recirculating system that reliably delivered adequate oxygen and managed *Pythium* risk throughout a six-month fruiting cycle would resolve both constraints simultaneously and permanently, rather than substituting one substrate input for another with a different but still meaningful environmental footprint. The scientific investment required to close the identified gaps is modest in absolute terms; the agronomic, economic, and ecological return on that investment, if the system can be made to work, is substantial.

This review has a boundary that should be stated explicitly. It synthesizes evidence from hydroponic systems engineering, plant physiology, and oomycete pathology to identify the constraints and research gaps relevant to NFT strawberry, but it cannot resolve whether those constraints are surmountable on a commercial scale under the economic conditions facing strawberry producers. That question depends on whether the dissolved oxygen and *Pythium* thresholds, once established, reveal engineering solutions that are economically viable within strawberry production margins, or whether the biological requirements of this crop in this system impose costs that no feasible intervention can bring within range. The answer is not knowable without the experimental sequence this review identifies. What can be stated with confidence is that the failure of NFT strawberry in the 1980s reflected real and unresolved biological constraints, not a premature rejection of a viable technology, and that any rational reconsideration of the system must begin with the quantitative characterization of those constraints rather than with optimistic extrapolation from more tractable crops.
